# Nutraceuticals in Parkinson’s Disease

**DOI:** 10.1007/s12017-016-8398-6

**Published:** 2016-05-04

**Authors:** Liting Hang, Adeline Henry Basil, Kah-Leong Lim

**Affiliations:** 1 Neurodegeneration Research Laboratory, National Neuroscience Institute, 11, Jalan Tan Tock Seng, Singapore, 308433 Singapore; 2 Department of Physiology, National University of Singapore, Singapore, Singapore; 3 NUS Graduate School for Integrative Sciences and Engineering, Singapore, Singapore; 4 Duke-NUS Medical School, Singapore, Singapore

**Keywords:** AMPK, Mitochondria, Parkin, Parkinson’s disease, Neurodegeneration

## Abstract

Current pharmacological strategies for Parkinson’s disease (PD), the most common neurological movement disorder worldwide, are predominantly symptom relieving and are often plagued with undesirable side effects after prolonged treatment. Despite this, they remain as the mainstay treatment for PD due to the lack of better alternatives. Nutraceuticals are compounds derived from natural food sources that have certain therapeutic value and the advent of which has opened doors to the use of alternative strategies to tackle neurodegenerative diseases such as PD. Notably, nutraceuticals are able to position themselves as a “safer” strategy due to the fact that they are naturally derived compounds, therefore possibly having less side effects. Significant efforts have been put into better comprehending the role of nutraceuticals in PD, and we will look at some of them in this review. Broadly speaking, these compounds execute their positive effects via modulating signalling pathways, inhibiting oxidative stress, inflammation and apoptosis, as well as regulating mitochondrial homoeostasis. Importantly, we will highlight how a component of green tea, epigallocatechin-3-gallate (EGCG), confers neuroprotection in PD via its ability to activate AMP kinase and articulate how its beneficial effects in PD are possibly due to enhancing mitochondrial quality control.

## Introduction

Parkinson’s disease (PD) is the most common neurodegenerative movement disorder currently affecting around 5–6 million predominantly elderly individuals worldwide. PD is expected to be even more commonplace in the near future as the world’s population rapidly ages. In 2030, about 10 million or more are expected to be afflicted with PD (Dorsey et al. 2007). Clinically, the disease is characterized by a constellation of motoric deficits including resting tremor, bradykinesia (slowness in movements), postural instability and rigidity that arises from the depletion of striatal dopamine—a result of the progressive loss of midbrain dopaminergic neurons in the substantia nigra pars compacta (SNpc) that innervate the striatum. This is accompanied by the characteristic neuropathological pattern of eosinophilic intracytoplasmic inclusions known as Lewy bodies (LBs) in surviving neurons in the SN. Notably, α-synuclein, a presynaptic protein whose mutations are causative of familial PD, is a major component of LBs (Polymeropoulos et al. 1997). Although a small percentage of PD cases are inheritable as a result of mutations in genes including *α*-*synuclein*, *Parkin*, *LRRK2*, *PINK1*, and *DJ*-*1*, exposure to environmental toxins and pesticides, such as paraquat and rotenone, and synthetic toxins, such as 1-methyl-4-phenyl-1,2,3,6-tetrahydropyridine (MPTP), can also lead to PD (Goldman 2014). To date, the exact disease mechanisms underlying PD pathogenesis are not fully understood, but studies have consistently implicated aberrant mitochondrial and protein homoeostasis as key contributors to the development of PD, with oxidative stress likely acting as an important nexus (Lim and Zhang 2013).

At present, therapeutic strategies for the PD patient remain largely symptomatic and more often than not, existing pharmacological treatments come with undesirable side effects. Indeed, pharmacological replacement of dopamine with l-Dopa remains as the gold standard for PD treatment despite its association with diminishing effects and problematic drug-induced dyskinesia after prolonged intake. These inadequacies of the gold standard treatment highlight an urgent need to develop more effective disease-modifying drugs for PD. In recent years, several alternative approaches to delay the progression of the disease have been considered and coming under the spotlight recently is nutraceuticals. Functional foods and nutritional supplements, which are common sources of nutraceuticals, are beginning to gain international recognition due to the potential health benefits they harbour when consumed as part of a varied diet on a regular basis and at optimal levels. Consequently, both the scientific community and food industry are motivated to exploit these benefits for the prevention and even treatment of chronic ageing diseases, including PD.

## Nutraceuticals and PD

As the word suggests, “nutraceuticals” refers to compounds that are derived from natural sources, and these food or derivatives therein have been clinically substantiated with reasonable scientific evidence to support their beneficial role in the prevention and/or treatment of a particular disease (Chao et al. 2012). It is this very reason that nutraceuticals are more readily accepted by the general populace as a form of treatment. There is a greater tendency to believe that there are fewer side effects associated with compounds derived from natural sources as compared to the many known side effects of synthetic drug compounds.

**Fig. 1:**
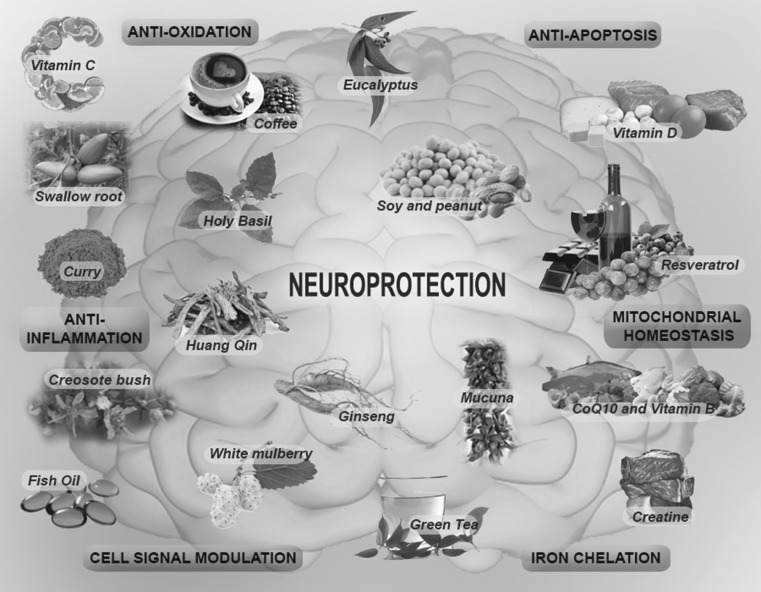
Nutraceuticals as therapeutics for PD. Nutraceuticals for PD can be grouped broadly into six themes based on their neuroprotective properties: (1) iron chelation; (2) cell signalling modulation; (3) anti-inflammation; (4) anti-oxidation; (5) anti-apoptosis; and (6) mitochondrial homoeostasis. However, several nutraceuticals hold multiple properties and function via a myriad of mechanistic pathways rather than adhere to a single mechanism

In the context of PD, several nutraceuticals have been shown to provide neuroprotection in experimental models and may serve as alternatives to synthetic drug compounds like l-Dopa that is known to cause many undesirable side effects. The mechanisms by which they work can be broadly classified into the following themes: (1) reactive oxygen species (ROS)/free radical scavenging; (2) anti-inflammation; (3) iron chelation; (4) modulation of cell signalling pathways; (5) anti-apoptosis; and (6) mitochondrial homoeostasis, although several nutraceuticals essentially function via a myriad of mechanistic pathways rather than adhere to a single mechanism (Fig. 1). Table 1 summarizes existing nutraceuticals that have been found to confer neuroprotection in PD. We will discuss a few examples of these below.

**Table 1:** Summary of nutraceuticals in PD

Nutraceuticals	Compound	Proposed mechanism of action	Evidence on potential neuroprotective effects			References
			Clinical trials	In vivo	In vitro	
Vitamin B complex	Vitamin supplement	Regulate levels of homocysteine Optimize mitochondrial function	PD patients treated with l-Dopa and vitamin B showed a reduction in homocysteine levels compared to without vitamin B.	Protect against MPTP-induced SNpc dopaminergic neuronal loss and striatal DA depletion in mice		Miller et al. (2003), Lamberti et al. (2005), Yokoyama et al. (2010), Anderson et al. (2008), Mukherjee et al. (1997)
Vitamin C and E	Antioxidant vitamin supplements	Vitamin C: Free radical scavenger in the cytosol Vitamin E: Lipid-soluble antioxidant to prevent lipid peroxidation in membranes	Conflicting reports			Fahn (1991), Martin et al. (2002), Parkinson Study Group (1993), Olanow (2003), Zhang et al. (2002), Etminan et al. (2005)
Vitamin D	Vitamin supplement	Upregulate GDNF levels (promote outgrowth of dopaminergic axons) Increase glutathione levels Calcium homoeostasis Anti-apoptotic Immunomodulatory effects Reduce nitric oxide synthase Regulate dopamine levels	Higher vitamin D serum levels, significantly lower risk of developing PD later in life	Attenuate 6-OHDA-induced and MPP+-induced neurotoxicity in rodent model Vitamin D receptor knockout mice developed motor defect		Wang et al. (2001), Knekt et al. (2010), Kirik et al. (2001), Garcion et al. (2002), Evatt et al. (2008)
Coenzyme Q10 (CoQ10)	Fat-soluble and vitamin-like quinone found abundantly in liver and the brain	Maintain proper transfer of electrons in the electron transport chain of mitochondria and ATP production Potent antioxidant that can reduce oxidized form of alpha-tocopherol to prevent lipid peroxidation	Conflicting reports	Attenuate MPTP-induced neurotoxicity in rodent model		Beal et al. (1998), Cleren et al. (2008), Shults et al. (2002), Muller et al. (2003), Shults (2005)
Creatine	Nitrogenous organic acid	Phosphorylated by creatine kinase to form phosphocreatine, an energy reserve in the brain and skeletal muscles Phosphocreatine is a key player in the maintenance of ATP levels, which in turn are important in synaptic activity and skeletal muscle functions	Creatine treatment improved mood and reduced the dosages required for dopamine replacement therapy	Reduce loss of dopaminergic neurons in MPTP mouse model		Matthews et al. (1999), Bender et al. (2006)
Fish oil	Polyunsaturated fatty acids (n-3 PUFA)	Important modulators for dopaminergic neurons in the basal ganglia Antidepressant effects mediated by an increase in serotonergic neurotransmission		Reduce dopamine loss and prevent formation of DOPAC in MPTP-induced parkinsonism in mice Calon F et al. (2007)		Denny Joseph and Muralidhara (2015), de Lau et al. (2005), Chen et al. (2003), Bousquet et al. (2008), Garcia-Arencibia et al. (2009)
*Mucunapruriens, Mucunasanjappae*	Natural sources of l-Dopa—plants belonging to *Mucuna* genus family	Consists of significant amounts of NADH and CoQ10 NADH: increases dopamine levels via upregulation of TH), counteracts the inhibition of mitochondrial complex 1 activity) and protects neurons against DNA damage caused by free radicals produced during the interaction between l-Dopa and divalent copper ions	Patients prescribed with mucuna seed powder extract showed significant improvements with better tolerability compared to l-Dopa treatment alone Importantly, in the mucuna-treated patients, severe dyskinesia or peripheral dopaminergic neuronal damage was observed	Protects against 6-OHDA toxicity in rodent model		Vaidya et al. (1978), Nagashayana et al. (2000), HP-200 in Parkinson’s Disease Study Group (1995), Manyam et al. (2004), Tharakan et al. (2007), Spencer et al. (1994), Patil et al. (2015), Hussian and Manyam (1997), Katzenschlager et al. (2004)
EGCG in green tea	Polyphenolic compounds	Passes BBB Iron chelation Free radical scavenger Antioxidant Regulation of PKC Modulation of ROS–NO pathway AMPK activation	Prospective cohort study of Singapore Chinese Health Study showed no relationship between green tea consumption and PD risk	Protects against 6-OHDA- and MPTP-induced parkinsonism in mice	Protects against 6-OHDA-induced toxicity in PC12 cells Protects against TNF-α and hydrogen peroxide-induced apoptosis in rat mesencephalic cells	Ng, Guan, et al. (2012), Pan et al. (2003), Mandel et al. (2006), Levites et al. (2001), Mandel et al. (2004), Guo et al. (2007), Tan et al. (2008), Xu et al. (2015)
Curcuminoids in curry	Polyphenolic flavonoid that constitutes approximately 4 % of turmeric	Antioxidant Restoring glutathione levels (protect neurons against protein oxidation and preserving mitochondrial complex 1 activity) Anti-inflammatory (inhibits LPS-induced morphological changes of microglia and reduces LPS-induced production of pro-inflammatory factors and their gene expressions)		Protect neurons against protein oxidation and preserve mitochondrial complex I activity Reduce 6-OHDA-induced neurotoxicity in rodent models Significantly reversed MPTP-induced depletion of DA and DOPAC in mice	Protect neurons against protein oxidation and preserve mitochondrial complex I activity Reduce 6-OHDA-induced neurotoxicity in MES 23.5 cells by modulating NF-κB translocation Prevent MPTP-induced neurotoxicity in SH-SY5Y cells and PC12 cells by targeting the JNK, the Bcl-2 mitochondria and the ROS–iNOS pathways	Jagatha et al. (2008), Zbarsky et al. (2005), Wang et al. (2009a), Yang et al. (2008), Yu et al. (2010), Chen et al. (2006), Rajeswari and Sabesan (2008), Wang et al. (2010), Pandey et al. (2008)
Baicalein	Flavonoid extracted from the root of *Scutellariabaicalensis*, a traditional Chinese herb commonly known as Huang Qin	Antioxidant Anti-inflammatory Inhibit fibrillization of alpha-synuclein		Rescues MPTP- and 6-OHDA-induced neurotoxicity in rodent models	Attenuate mitochondrial depolarization and proteasome inhibition in PC12 cells induced by familial alpha-synuclein mutation (E46K).	van Leyen et al. (2006), Chen et al. (2008), Cheng et al. (2008), Mu et al. (2009), Waxman et al. (2010), Jiang et al. (2010)
Resveratrol	A phytoalexin found in plants such as grapes, peanuts, berries and pines	Inhibition of NADPH oxidase and suppression of IL1-a and TNF-α triggered by LPS Modulate levels of BAX and Bcl-2 in vitro Stimulation of SIRT1 in SK-N-BE cells Free radical scavenger		Attenuate MPTP-, 6-OHDA- and LPS-induced toxicity in rodent models	Protect dopaminergic neurons against toxicity induced by LPS, DA or MPTP	Zhang et al. (2010), Bournival et al. (2009), Lee et al. (2007), Lu et al. (2008), Khan et al. (2010), Bureau et al. (2008), Albani et al. (2009), Pallas et al. (2009), Pallas et al. (2008)
Oxyresveratrol	Heartwood or fruit of *Artocarpusheterophyllus, Artocarpuslakoocha, Actocarpusgomezianus and Actocarpusdadah* Wood or fruit of mulberry trees Fruit of *Melaleucaleucadendron* Rhizomes of *Smilacischinae* Egyptian herb *Schoenocaulonofficinale*	Anti-inflammatory effects, particularly those isolated from *Artocarpusheterophyllus, Artocarpusdadah*or mulberry wood Free radical scavenger (penetrates neurons easily) Upregulate SIRT1			Protect against 6-OHDA-induced toxicity in SH-SY5Y cells by reducing the release of lactate dehydrogenase and caspase-3-specific activity	Fang et al. (2008), Su et al. 2002), Chao et al. (2008)
Ginsenoside	Ginsenosides, a phytoestrogen, are a class of molecules extracted from several species of ginseng	Regulate several pathways (P13K/AKT, ERK, JNK, ROS-NFkB, IGF-1 receptor signalling pathways and oestrogen receptor pathway) Maintain glutathione levels Anti-apoptotic (Attenuate JNK signalling) Prevent elevation of iron levels by regulating the expression of iron transport proteins		Attenuate MPTP-, 6-OHDA- and rotenone-induced toxicity in rodent models		Chen et al. (2005), Wang et al. (2009b), Xu et al. (2009), Leppa and Bohmann (1999)
Genistein	A phytoestrogen found mainly in soy and peanuts	Binds to oestrogen receptor β and upregulates anti-oxidative and anti-apoptotic genes Antioxidant (Increase the levels of malondialdehyde, superoxide dismutase, and monoamine oxidase) Anti-apoptotic (Tyrosine kinase inhibitor, attenuate activation of PKC)		Attenuate 6-OHDA-induced toxicity in rodents	Attenuate hydrogen peroxide-induced cell death in N27 cells	Kaul et al. (2005), Baluchnejadmojarad et al. (2009)
Holy Basil (*Ocimum sanctum*)	A leaf extract from a plant known as Tulsi, which is found throughout India	Anti-microbial Anti-stress Anti-diabetic Hepatoprotective Anti-inflammatory Neuroprotective Cardioprotective		Delay loss of climbing ability and reduce the oxidative stress in brain of the *Drosophila* PD model		Siddique et al. (2014)
Nucleoprotein	Extracted from salmon soft roe and consists mainly of a mixture of DNA nucleotide and protamine	ROS scavenger Reduce accumulation of lipofuscin-like substances in the brain, which is often related to Lewy body formation		Prevent locomotor impairment and dopaminergic neuronal degeneration in MPTP-induced toxicity mice model		Kiriyama et al. (2015)
Nordihydroguaiaretic acid (NDGA)	Polyphenol extracted from compound of creosote bush (*Larrea tridentate*)	Antioxidant Anti-genotoxic Anti-neoplastic Antiviral Anti-inflammatory Inhibit the accumulation of alpha-synuclein (hallmark of PD)		Delay the loss of climbing ability in Drosophila PD model		Siddique et al. (2012)
Quercetin (Q)	Natural flavonoid found in fruits and vegetables such as onion, broccoli and apple	Antioxidant Anti-inflammatory Anti-cancer		Protect against chronic rotenone toxicity and dopaminergic neuronal loss in 6-OHDA rat models of PD		Haleagrahara et al. (2011), Denny Joseph and Muralidhara (2015)
Magnesium	Dietary supplement	DA uptake Vesicular storage and transport Alter Ca^2+^-mediated neurotoxicity Activate CuZn-SOD, thereby attenuating formation of ONOO–, involved with α-synuclein aggregation				Philippu et al. (1975), Safar et al. (2010), Lin et al. (2002), Johnson (2001)
Caffeine	Psychoactive CNS stimulant found in coffee	Regulate expression of genes involved in oxidative stress (cytochrome c oxidase subunits, enolase alpha, NADH dehydrogenase, aldehyde dehydrogenase) Regulate expression of ubiquitin–proteasome pathway-related genes (ubiquitin-conjugating enzyme, protease 26S subunit, ubiquitin B and C) Cell-cycle regulation	Risk of suffering PD decreases as consumption of coffee increases	Protect against 6-OHDA- and MPTP-induced dopaminergic neuronal loss in mice		Gongora-Alfaro (2010), Singh et al. (2010)
Eucalyptus Oil	Eucalyptus citriodora leaf extract	Antioxidant		Delay of the loss of climbing ability in *Drosophila* PD model		Siddique et al. (2013)
*Ginkobiloba* extract (EGb 761)	*Ginkobiloba* leaves containing flavonoids and terpenoids	Antioxidant Anti-inflammatory Anti-apoptotic		Pretreatment with EGb 761 decreases lipid peroxidation and improves locomotor activity in 6-OHDA rat model In a MPTP rat model, EGb 761 prevents dopaminergic neurotoxicity, decreases SOD activity, decreases oxidative stress and apoptosis induced by MPP+	EGb 761 provide neuroprotection against paraquat-induced apoptosis of PC12 cells	Kim et al. (2004), Kang et al. (2007), Rojas et al. (2012), Ahmad et al. (2005), Yang et al. (2001)
Shengmai San (SMS) and LingGuiZhuGanTang (LGZGT)	Traditional Chinese Medicine SMS comprise of three crude drug components, Radix Ginseng (Panax ginseng) (Araliaceae), Radix Ophiopogonis (Ophiopogonjaponicus) (Liliaceae) and FructusSchisandrae (Schisandrachinensis) (Schisandraceae) LGZGT comprise of four crude drug components, Cinnamon twig (Cinnamomum cassia Presl.) (Lauraceae), Atractylodisrhizoma (AtractylodesmacrocephalaKoidz.) (Compositae), Glycyrrhizae radix (GlycyrrhizauralensisFisch.) (Leguminosae) and one fungi Hoelen (Poriacocos (Schw.) (Wolf Polyporaceae)	Antioxidant		Protects against MPTP-induced dopaminergic neuronal loss in mice		Giridharan et al. (2011)
Swallow root *(Decalepishamiltonii)*	Extract from plant species swallow root	Antioxidant		Improved climbing ability and circadian rhythm of locomotor activity of transgenic alpha-synuclein *Drosophila* PD model and resulted in an associated reduction in levels of ROS and lipid peroxidation and enhancement in the activities of catalase and superoxide dismutase		Jahromi et al. (2015)
TianmaGouteng Yin (TGY)	Traditional Chinese Medicine	Unclear		Improved survival rates and locomotor function of rotenone-intoxicated *Drosophila* PD model Reduced levels of alpha-synuclein and prevented degeneration of dopaminergic neurons in alpha-synuclein transgenic *Drosophila* Protects rotenone-induced dopaminergic neuronal loss in mice	Alleviated apoptotic cell death in human dopaminergic neuroblastoma SH-SY5Y cell line treated with rotenone	Liu et al. (2015)

The seeds of the *Mucuna* plant, also affectionately known as dopa bean, are well known for containing l-Dopa, the go-to drug for treating PD. Although some species of *Mucuna* contain more l-Dopa than others, the *Mucuna* plant is generally favoured for the exploitation of l-Dopa due to its relative abundance of which compared to other plant families that have been studied (Patil et al. 2015). Other microbial and chemical means of synthesizing l-Dopa have also been explored (Surwase et al. 2012; Krishnaveni et al. 2009; Ali et al. 2007; Sikander and Ikram ul 2006), but the *Mucuna* plant has been preferred as it is a natural and inexpensive source, and it provides additional benefits as an antioxidant (Manyam et al. 2004). In fact, a species of *Mucuna* plant, *Mucuna pruriens*, has been shown in both the PD mice model and patients to be more effective than l-Dopa without the accompanying increase in dyskinesia (Hussian and Manyam 1997; Katzenschlager et al. 2004).

Apart from the *Mucuna* plants, there are many other nutraceuticals that appear to be neuroprotective due to their anti-oxidative properties. Such properties are particularly important in the context of PD as several studies have pointed to oxidative stress, which results in ROS generation and inflammation, as a pivotal contributor to age-related neuronal loss in PD (Jenner 1998). An example of a nutraceutical that possesses both anti-oxidative and anti-inflammatory properties is ginsenoside, a phytoestrogen that is extracted from several species of ginseng (Chen et al. 2005). It executes its anti-oxidative properties by maintaining glutathione levels, and its anti-inflammatory properties are a result of the regulation of several inflammatory pathways including the ROS-NFκB, JNK, P13K/AKT, ERK, IGF-1 receptor signalling pathways and oestrogen receptor pathway. In addition, ginsenoside also reduces the levels of nigral iron of MPTP-treated mice by regulating the expression of iron transport proteins (Wang et al. 2009b). This is of importance as the build-up of iron in conjunction with ROS at the site of neurodegeneration is thought to constitute a major trigger in neurotoxicity and neuronal demise in PD (Zecca et al. 2004). As such, nutraceuticals like ginsenoside that can inhibit pro-inflammatory and oxidative processes should, in theory, be able to attenuate dopaminergic neuronal damage. Indeed, it has been demonstrated that ginsenoside protects against toxicities and dopaminergic neuronal loss induced by PD toxins including 6-hydroxydopamine (6-OHDA) and MPTP (Chen et al. 2005; Xu et al. 2009). Due to its role in the regulation of JNK signalling, ginsenoside also possesses anti-apoptotic properties. Hence, another postulated mechanism through which the neuroprotective effect of ginsenoside is facilitated is its reduction of c-Jun phosphorylation, which prevents pro-apoptotic JNK signalling and dopaminergic neuronal loss during MPTP-induced neurotoxicity (Leppa and Bohmann 1999).

Besides ginseng, dietary soy and peanut products have also been reported to have similar anti-apoptotic effects. Soy and peanut are rich sources of genistein, a phytoestrogen-like ginsenoside. Genistein acts as a tyrosine kinase inhibitor that attenuates protein kinase C (PKC) activation and thereby downstream apoptotic effects (Kaul et al. 2005; Baluchnejadmojarad et al. 2009). Another potent anti-apoptotic nutraceutical that has been shown to protect against PD toxin-induced neurotoxicity is *Ginkobiloba* extract EGb 761. EGb 761 prevents the formation of apoptosome and the apoptotic cascade by blocking cytochrome-c release (Liu et al. 2008; Yeh et al. 2009; Nevado et al. 2010). Like ginsenoside, EGb 761 also attenuates the phosphorylation of c-Jun (Shi et al. 2009) and furthermore inhibits the cleavage of caspase-3 (Liu et al. 2008; Shi et al. 2009), thereby preventing DNA fragmentation, a hallmark of apoptosis. By blocking apoptosis through various mechanistic pathways, genistein and EGb 761 were found to attenuate dopaminergic neuronal loss and reduce associated locomotion impairment in 6-OHDA and MPTP mice models (Ahmad et al. 2005; Baluchnejadmojarad et al. 2009; Rojas et al. 2012; Yang et al. 2001).

## Nutraceuticals and Mitochondrial Homoeostasis

As mentioned earlier, aberrant mitochondrial homoeostasis is commonly implicated in PD pathogenesis. Intuitively, one would propose that nutraceuticals that have a role in mitochondrial regulation can potentially mitigate PD pathology. Coenzyme Q10 (CoQ_10_) is a component of the mitochondrial electron transport chain and participates actively in ATP generation. It is noteworthy to mention that in PD animal models, CoQ_10_ attenuates MPTP-induced neurotoxicity, possibly due to its unique electron-accepting property, rendering it critical to the electron transfer between mitochondrial complex 1 and other complexes of the electron transport chain (Beal et al. 1998; Cleren et al. 2008). Although it has been proposed as a therapeutic strategy for PD (Shults 2005), clinical trials involving CoQ_10_ have been conflicting. While one study by Shults et al. reported a dose-dependent reduction in functional decline, another study by Muller et al. observed only mild symptomatic benefit (Shults et al. 2002; Muller et al. 2003). Other nutraceuticals that are reported to be neuroprotective due to their role in preserving mitochondrial complex 1 activity include curcuminoids from turmeric (Jagatha et al. 2008) and the earlier-mentioned *Mucuna* plant (Manyam et al. 2004). Yet another key player in the maintenance of ATP levels is phosphocreatine, an energy reserve in skeletal muscles and brain. Notably, treatment with creatine appears to rescue parkinsonian phenotypes in both human subjects and animal models. Specifically, diet supplement of creatine was found to improve the mood and reduce the dosages required for dopamine (DA) replacement therapy in PD patients (Bender et al. 2006), as well as reduce dopaminergic neuronal loss in SNpc of MPTP-treated mice (Matthews et al. 1999). It certainly seems that mitochondrial homoeostasis is a common targeted pathway for nutraceutical therapy notwithstanding the controversy surrounding CoQ_10_.

Epigallocatechin-3-gallate (EGCG), a main green tea-derived catechin, is a nutraceutical that is frequently featured in PD, perhaps due to its numerous putative neuroprotective mechanisms that is not limited to mitochondrial homoeostasis (Pan et al. 2003). These include anti-oxidation, iron chelation, ROS scavenging and anti-apoptotic properties. Moreover, EGCG crosses the blood–brain barrier easily, making it an attractive compound for therapy. Besides the myriad of properties, EGCG has been reported to be an AMPK activator (Spasic et al. 2009; Hwang et al. 2009). It increases cytosolic Ca^2+^ levels, thereby influencing the activity of Ca^2+^-/calmodulin-dependent protein kinase kinase (CaMKKβ), an upstream kinase of AMPK (Kim et al. 2014). The activation of AMPK in the presence of EGCG is therefore likely to be mediated by CaMKKβ. As discussed below, AMPK activation by EGCG has been demonstrated to be neuroprotective.

## AMPK Activation and Neuroprotection

AMPK is a central energy sensor and regulator that is normally activated in response to diminishing energy supply, e.g. ATP depletion or glucose starvation (Li et al. 2012). Given the critical role of AMPK in energy homoeostasis, it is perhaps not surprising to note that AMPK has profound influence on mitochondrial homoeostasis amidst a plethora of metabolic events that it governs. It is well documented that AMPK works through peroxisome proliferator-activated receptor gamma coactivator 1-alpha (PGC-1α) to promote mitochondrial biogenesis (Lee et al. 2012). Although the mechanism by which AMPK upregulates PGC-1α activity remains unclear, studies have suggested that it could directly phosphorylate PGC-1α (Ng et al. 2012b) or indirectly activate the transcriptional coactivator by promoting its deacetylation through the NAD^+^-dependent deacetylase SIRT1 (Ng et al. 2012b). Interestingly, a recently identified AMPK target is UNC-51-like kinase 1 (ULK1), a mammalian ortholog of the yeast Atg1 kinase that acts as a key initiator of the autophagy cascade (Carroll et al. 2014; Martin et al. 2014). The activation of ULK1 by AMPK promotes autophagy, including mitophagy. Accordingly, when ULK1 function is impaired, it results in the accumulation of abnormal mitochondria with reduced potential (Martin et al. 2014). Similarly, when the known AMPK-mediated phosphorylation sites on ULK1 are abolished, autophagy is also impaired (Martin et al. 2014), suggesting that the clearance of damaged mitochondria is dependent on the AMPK–ULK1–autophagy pathway. More recently, a study by Toyama et al. reported that AMPK is required for rotenone-induced mitochondrial fission (Toyama et al. 2016), an essential process to isolate damaged mitochondria and promote mitophagy (Twig et al. 2008). Importantly, they identified a novel substrate of AMPK, mitochondrial fission factor (MFF), and found that the presence of non-phosphorylatable MFF resulted in defective mitochondrial fission, further emphasizing the importance of AMPK in regulating mitophagy.

Notwithstanding earlier discussion that AMPK activation helps to maintain mitochondrial quality control and should theoretically promote cellular survival, the role of AMPK activation in neuroprotection remains controversial. Similarly, AMPK activation is also a double-edge sword in the case of PD, promoting neurodegeneration under some circumstances yet aggravating in others. For instance, a study by Kim et al. contradicts a neuroprotective role of AMPK in PD and found that AMPK mediates dopaminergic neuronal atrophy in 6-OHDA-lesioned mice. Moreover, metformin-induced AMPK activation accelerates rather than retards 6-OHDA-induced neuronal loss in these mice (Kim et al. 2013). Subsequently, Xu et al. also observed in a related study similar detrimental effects of AMPK activation in primary neurons treated with 6-OHDA, MPTP or rotenone (Xu et al. 2014). Nonetheless, there are also several reports supporting a neuroprotective role of AMPK in PD. In a recent study by Patil et al., MPTP-treated mice on chronic metformin regimen demonstrated enhanced antioxidant activity and brain-derived neurotrophic factor (BDNF) levels, thereby rendering protection against dopaminergic neuronal loss induced by MPP+ (Patil et al. 2015). In a related study, AMPK is activated upon MPTP treatment, and when AMPK activity is downregulated by compound C, neurotoxicity is enhanced (Choi et al. 2010). Supporting this, a recent study demonstrated similar findings albeit in cultured cells exposed to rotenone (Wu et al. 2011). Given that both MPP + and rotenone are complex I inhibitors, the rescue of the in vivo and in vitro PD models by AMPK activation is consistent with its role in the maintenance of mitochondrial homoeostasis. Importantly, it is also noteworthy to mention that a recent cohort-based study involving 800,000 subjects in a Taiwanese population revealed that metformin-inclusive sulfonylurea therapy significantly reduces PD risk in individuals with type 2 diabetes (Wahlqvist et al. 2012), suggesting the neuroprotective effects of AMPK activation. Collectively, these findings suggest that AMPK activation may be beneficial for the disease.

On a related note, we have recently found that EGCG facilitates neuroprotection in PD by mediating mitochondrial regulation via AMPK activation. Using *Drosophila* as a model, we found that EGCG administration ameliorates the pathological phenotypes of parkin null PD flies, including prominent mitochondrial abnormalities and progressive loss of selected dopaminergic neuronal clusters that are accompanied by an age-dependent decline in locomotor ability (Ng et al. 2012a). These are disease phenotypes that bear resemblance to that of human PD (Green and Kroemer 2004; Whitworth et al. 2005). Importantly, the EGCG-mediated protective effects require AMPK as genetic inactivation of AMPK abolishes the neuroprotective effects while subsequent genetic restoration of AMPK and pharmacological activation of AMPK with potent AMPK activators (metformin or AICAR) reproduce these beneficial effects (Ng et al. 2012a). In contrast, treatment of parkin null flies with another compound, i.e. Baicalein—an established antioxidant (Shieh et al. 2000), failed to ameliorate the observed parkinsonian phenotypes (unpublished observation). Accordingly, we speculated that AMPK activation rather than anti-oxidation may be involved in EGCG-mediated protective effects. In a similar fashion, *Drosophila* LRRK2 mutants could be rid of its pathological phenotypes via pharmacological treatment with EGCG, metformin or AICAR or the co-expression of a constitutively active AMPK mutant (Ng et al. 2012a), suggesting that this approach may be relevant to different forms of PD. Consistent with our results, Ferretta et al. demonstrated similar benefits using resveratrol (Ferretta et al. 2014), another nutraceutical found in the skin of grapes and berries that is known to be a relatively strong AMPK activator (Dasgupta and Milbrandt 2007). In patient’s fibroblasts harbouring parkin mutations, they found that resveratrol increased mitochondrial biogenesis and improved oxidative phosphorylation (Ferretta et al. 2014).While AMPK activation brings about a virtually complete rescue of PD pathological phenotypes in flies, we are currently on the endeavour to dissect precisely how it happens. Nevertheless, existing knowledge of AMPK has shed light on the possible mechanistic pathways via which AMPK-mediated neuroprotection may occur. As mentioned earlier, AMPK is capable of positively regulating mitochondrial biogenesis via PGC-1α and also mitophagy. This is further supported by the resveratrol-based study by Ferretta et al. that also revealed the ability of AMPK to enhance autophagy flux in parkin-mutant fibroblasts (Ferretta et al. 2014). Hence, it is likely that enhanced mitochondrial biogenesis and/or mitophagy could help in the maintenance of a viable pool of bioenergetically competent mitochondria necessary for dopaminergic neuronal survival. Accordingly, approaches towards promoting these processes may be of therapeutic value for PD.

## Conclusion

As with many other neurodegenerative diseases, PD is a debilitating disorder that gradually robs an individual of his/her fundamental bodily functions. Although much effort has been put into advancing therapeutic strategies for this disease, many conventional and existing treatment options are unfortunately accompanied by several undesirable effects despite their ability to provide symptomatic relief. Fortunately, with the advent of nutraceuticals, an alternative avenue to tackle this seemingly evasive biological problem has been provided. Nutraceuticals, by virtue of their origin from naturally available food or food products, appear to be a favourable treatment option since harnessing therapeutic strategies from natural resources can potentially avoid side effects. As a matter of fact, many of the nutraceuticals discussed in this review have been shown to be not only preventive but also therapeutic for PD. Significantly, we have highlighted in this paper how we and others have demonstrated the ability of green tea-derived catechin EGCG to rescue PD pathological outcomes, possibly through enhancing mitochondrial homoeostasis. However, despite many promising reports about the role of nutraceuticals in neuroprotection for PD, we acknowledged that it is early days yet as several mechanistic gaps remain unanswered. Notwithstanding this, the recognition that nutraceuticals might be of therapeutic benefits offers countless opportunities to explore other natural compounds that have not been looked at in terms of their potential neuroprotective roles in PD. For example, ergothioneine (EGT) is a naturally occurring amino acid found in mushrooms that protects mitochondria from oxidative stress (Cheah and Halliwell 2012) and was found to accumulate at significantly lower levels in PD patients compared to healthy controls (Hatano et al. 2015), thus suggesting a therapeutic potential of EGT for the disease. In addition, future research could also direct efforts towards better understanding the effects of nutraceuticals in combination with existing drug therapies for PD patients, in order to derive improved outcomes for the PD patient. The delivery of nutraceuticals could also be optimized in order to maximize their neuroprotective effects. As a parting note, it is hopeful to envisage that one day we would be able to simply modify our diet to prevent or mitigate the progression of this debilitating disease.
